# Automated Modal Analysis for Tracking Structural Change during Construction and Operation Phases

**DOI:** 10.3390/s19040927

**Published:** 2019-02-22

**Authors:** Jun Teng, De-Hui Tang, Xiao Zhang, Wei-Hua Hu, Samir Said, Rolf. G. Rohrmann

**Affiliations:** 1 School of Civil and Environmental Engineering, Harbin Institute of Technology (Shenzhen), Shenzhen 518055, China; tengj@hit.edu.cn (J.T.); tangdehui@stu.hit.edu.cn (D.-H.T.); 16S055247@stu.hit.edu.cn (X.Z.); 2 Federal Institute for Materials Research and Testing (BAM), 12205 Berlin, Germany; samir.said@bam.de; 3 Struktur Analyse & Bauwerks Monitoring (SABM) GbR, 10965 Berlin, Germany; rolf.rohrmann@gmx.de

**Keywords:** automated modal analysis (AMA), system model order, density-based spatial clustering of applications with noise (DBSCAN), continuous dynamic monitoring, temperature effect

## Abstract

The automated modal analysis (AMA) technique has attracted significant interest over the last few years, because it can track variations in modal parameters and has the potential to detect structural changes. In this paper, an improved density-based spatial clustering of applications with noise (DBSCAN) is introduced to clean the abnormal poles in a stabilization diagram. Moreover, the optimal system model order is also discussed to obtain more stable poles. A numerical simulation and a full-scale experiment of an arch bridge are carried out to validate the effectiveness of the proposed algorithm. Subsequently, the continuous dynamic monitoring system of the bridge and the proposed algorithm are implemented to track the structural changes during the construction phase. Finally, the artificial neural network (ANN) is used to remove the temperature effect on modal frequencies so that a health index can be constructed under operational conditions.

## 1. Introduction

Recently, the status monitoring of full-scale structures by applying operational modal analysis (OMA) has become more attractive because it can characterize the structural behavior under operational conditions [1]. Peeters investigated the environmental effect on modal parameters of the Z24 bridge based on the OMA [2]. Filipe proposed the online automatic OMA and applied it to a long-span arch bridge [3]. Hu presented an automated OMA method to study the behavior of a stress-ribbon footbridge under operational conditions [4]. William applied the OMA technique to achieve a near real-time damage detection [5]. Furthermore, OMA is widely applied to identify the modal parameters of wind turbines [6,7]. The modal parameters of stadiums during a football game were also reported [8] and tracked to detect the structural changes [9].

Though the key algorithm of the OMA technique, such as the subspace stochastic identification (SSI) method [3], can be automated in order to track the evolution of modal parameters and detect structural changes under operational conditions [10,11,12,13], some obstacles still exist for a fully automated modal analysis (AMA) procedure. They are summarized as follows:

Regarding the SSI method, the modal parameters are identified by interpreting the stabilization diagram. This was constructed by identifying the state-space model with different orders. By comparing the poles corresponding to a certain model order with the poles of a one-order-low model, the stable poles are found and a stabilization diagram is efficiently constructed. An obvious obstacle of AMA is the interference of spurious modes in a stabilization diagram. Unlike mathematical poles, the physical poles stay around the consistent modal parameters with increasing model orders. Meanwhile, the mode shape components of a physical mode should lie on a straight line in the complex plane. Taking these characteristics into account, different modal validation criteria, such as modal assurance criteria (MAC) [14], modal energy level [15], mean phase deviation [16] and the modal phase collinearity (MPC) criteria [17] are introduced to clean the spurious poles. However, the spurious poles cannot be totally eliminated by only applying these cleaning criteria. Thus, the clustering algorithm, including hierarchical clustering [4], *k*-means clustering [16] and combination of both methods [3], are further used to clean the stabilization diagram. However, initial values and multi-iteration are necessary in both *k*-means clustering and hierarchical clustering, which leads to a relatively longer computation time. To overcome this difficulty, a density-based spatial clustering of applications with noise (DBSCAN) has been reported to be less time-consuming and more potent in the detection and removal of noise data in the field of mathematics [18]. Additionally, the determination of the radius and minimum points in DBSCAN has a significant impact on the clustering analysis of massive data [18,19].

In this paper, the DBSCAN algorithm is introduced to remove the spurious poles in a stabilization diagram, and an efficient parameter optimization method based on the Euclidean distance is developed to realize a full AMA procedure for civil engineering structures.

Besides, the system model order, namely, the size of the Toeplitz matrix in covariance-driven subspace stochastic identification (SSI-COV) [20] has a considerable impact on the accuracy and precision of modal parameter estimation [21]. Generally, the model order needs to be overestimated so that all the physical modes are identified. However, with an increasing model order, more mathematical poles will be produced in the stabilization diagram. The traditional way to choose the model order is to determine fixed parameters, artificially based on experience or a number of trials [22,23]. In the field of mathematics, Markovsky introduced the minimum norm of the given order to determine the model order [24]. Hu obtained the optimal order by the iterative Cadzow’s algorithm, which was verified by the measured impulse response function [25].

However, the above-mentioned methods are computationally expensive. In this paper, the minimum reduction rate of the Frobenius norm is proposed to determine the model order of the civil engineering structures.

Finally, modal frequency is inevitably influenced by temperature under operational conditions, which may explain the variation caused by structural changes in the early stages [5]. Principal component analysis [5] and polynomial regression (PR) [10] are applied to establish the nonlinear relationship between them. In this paper, the nonlinear relationship models, constructed by both artificial neural network (ANN) and PR, are compared. The optimal model is applied to eliminate the temperature effect, and the remaining error matrix is used as an index to detect the structural changes under operational conditions.

The main contributions of the current paper may be summarized in three parts: First, the full AMA algorithm is proposed. The minimum reduction rate of the Frobenius norm is used to determine the optimal model order. Then, the improved DBSCAN algorithm, with parameter optimization, is introduced to eliminate the spurious poles in the stabilization diagram. Subsequently, the AMA algorithm is validated by the modal experiment of an arch bridge, and furthermore, it is applied to track the variation of modal frequency, based on a continuous dynamic monitoring of an arch bridge. Finally, the temperature effect on frequency is removed by the ANN method. The remaining residuals are used to construct the damage sensitive indices.

## 2. Automated Modal Analysis Based on DBSCAN

### 2.1. Covariance Driven Stochastic Subspace Identification Algorithm

On the assumption that the structure is a linear and time-invariant system, SSI identification techniques are formulated using a discrete time stochastic state-space model, according to:

(1) $$x_{k+1}=Ax_{k}+w_{k}$$

(2) $$y_{k}=Cx_{k}+v_{k}$$

where $x_{k}$ is the discrete-time state vector with system order *n*; $y_{k}$ represents the output vector; and $w_{k}$ and $v_{k}$ (*k* = 1, ..., *N*, *N* is the number of sampling points) are the process noise and measurement noise vectors, respectively. Matrix ***A*** is the discrete state matrix, and ***C*** is the observation matrix.

The discrete state matrix and observation matrix are estimated by the Toeplitz matrix T that is constructed by covariance matrices in SSI-COV. Singular value decomposition (SVD) is applied to the Toeplitz matrix to remove the noise component by low-rank approximation.

(3) $$T=USV^{T}$$

(4) $$S={\left[\begin{array}{cc} S_{d\times d} & 0\\ 0 & 0 \end{array}\right]}_{j\times j}$$

where ***S*** is the diagonal matrix of the same dimension as ***T***, while ***U*** and ***V*** are the unitary matrices. *d* in Equation (4) represents the target model order, and *j* is the size of the Toeplitz matrix.

The natural frequencies $\omega_{i}$ and damping ratios $\xi_{i}$ are estimated by the eigenvalue of the estimated state matrix, while the mode shapes M are identified from the observation matrix and the eigenvectors Ψ:

(5) $$\omega_{i}=\left|\lambda_{i}\right|\frac{\left|\lambda_{i}\right|}{2\pi }$$

(6) $$\xi_{i}=\frac{-Re(\lambda_{i})}{\omega_{i}}$$

(7) M=CΨ

More details of the covariance driven stochastic subspace identification method are further illustrated in Ref. [20].

For the SSI method, the modal parameters are identified by interpreting the stabilization diagram. This was constructed by identifying the state-space model with different orders. By comparing the poles corresponding to a certain model order with the poles of a one-order-low model, the stable poles are found, and a stabilization diagram is efficiently constructed.

### 2.2. Determination of the Optimal Model Order

In practice, because there is not a clear drop in the decreasing singular values, the model order is usually over-estimated so that all eigenvalues of the state matrix are identified. However, a redundant model order will lead to more spurious poles in the stabilization diagram. Meanwhile, the identified modal parameters will be unstable when the model order is too large. The optimal model order should not only guarantee that all physical modes are identified, but also minimize the effect of noise.

In general, the error matrix between the original Toeplitz matrix T and the estimated one ${\tilde{T}}_{e}$ (Equation (8)) can be evaluated by the Frobenius norm ${\left\|T-{\tilde{T}}_{e}\right\|}_{2}$. Theoretically, the physical modes contribute more to the Toeplitz matrix than noise, thus, the Frobenius norm decreases as the model order grows larger. The variation tendency of the Frobenius norm is similar to the singular value, and it is difficult to define the accurate model order directly. In this paper, the logarithm of the reduction rate of the Frobenius norm (Equation (9)) is proposed to identify the model order, since it first decreases and then increases with an increasing model order. This means that the contribution of the physical modes dominates in the estimated Toeplitz matrix ${\tilde{T}}_{e}$, when the reduction rate of the Frobenius norm decreases at a relatively faster rate with the rising model order. After reaching the minimum value, the reduction rate of the Frobenius norm begins to increase gradually, reflecting that the contribution of the physical mode is covered by spurious poles and the identified modal parameters tend to be discrete because of the noise effect. Therefore, the accurate model order can be determined by the location of the minimum value of the logarithm of the reduction rate of the Frobenius norm (Equation (10)):

(8) $${\tilde{T}}_{e}=U_{j\times e}S_{e\times e}V_{e\times j}$$

(9) $$f(e)={log}_{10}(\frac{{\left\|T-{\tilde{T}}_{e-1}\right\|}_{2}-{\left\|T-{\tilde{T}}_{e}\right\|}_{2}}{{\left\|T-{\tilde{T}}_{e-1}\right\|}_{2}})\;(e=1,2,\ldots ,n)$$

(10) e=argmin(f(e))

**Figure 1:**
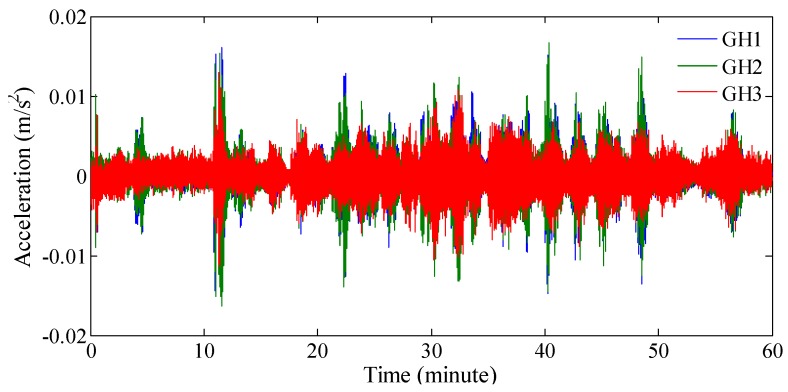
Typical responses recorded by three accelerometers in an arch.

The strategy for the determination of the model order can be explained further by the following case. Figure 1 shows the typical structural responses acquired from three accelerometers, installed on the arch of a bridge. The signals are recorded over the course of 10 min, with a sampling frequency of 20 Hz (more details can be found in Section 3).

**Figure 2:**
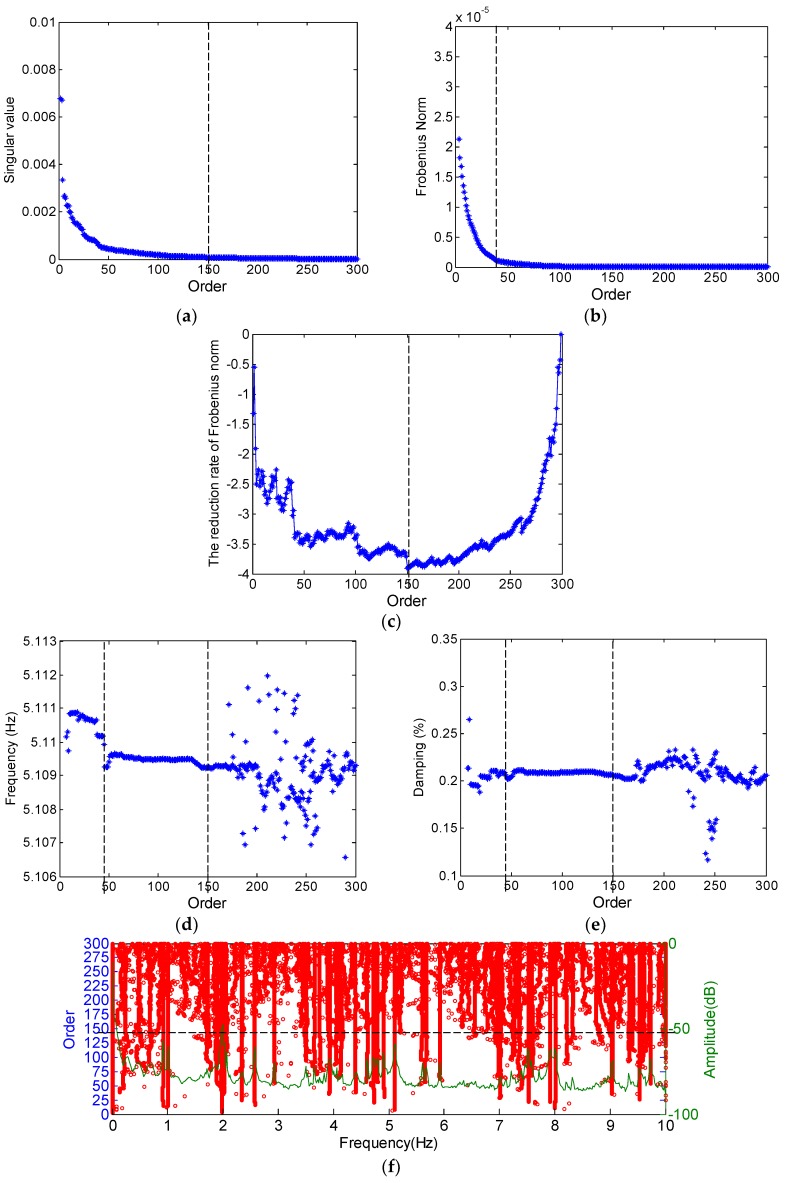
Determination of the model order: (**a**) singular value; (**b**) the Frobenius norm between the original and the estimated Toeplitz; (**c**) the reduction rate of the Frobenius norm; (**d**) identified frequencies; (**e**) identified damping ratio; and (**f**) identified poles by selecting the maximum order.

From Equations (3) and (4), the singular values of the Toeplitz matrix ***T*** are shown in Figure 2a. The corresponding Frobenius norm of the error matrix ${\left\|T-{\tilde{T}}_{e}\right\|}_{2}$ is plotted in Figure 2b. It can be observed from Figure 2a,b that both indices generally decrease with the rising model order. When the model order varies from 0 to 50, both indices drop significantly. However, when the model order falls in the range of 50 to 300, both indices approach zero gradually. Thus, it is difficult to directly define the model order from Figure 2a,b. Figure 2c displays the proposed reduction rate of the Frobenius norm f(e), according to Equations (9) and (10). It is found that f(e) decreases quickly, when the model order varies from 0 to 50, and then reaches the minimum gradually, at around model order 150. This suggests that the physical modes contribute more components than the noise in the Toeplitz matrix. Subsequently, f(e) begins to increase until the model order reaches 300. Based on the minimum value in the curve of f(e), the model order is selected as approximately 150 for the arch.

In order to validate the correctness of the proposed algorithm for model order selection, the frequency and damping of a vibration mode (5.11 Hz) in an un-truncated order are shown in Figure 2d,e, respectively. It can be seen that the identified modal frequency and damping ratio remain stable, when the model order changes from 50 to 150. When the model order goes beyond 150, the identification results spread gradually due to the effect of noise. Meanwhile, it is interesting to note that the estimates of both frequency and the damping ratio have relatively low precision, when the model order is below 50. This may partially result from the fact that some of the significant information is filtered out by SVD, when the model order is too small [20].

Furthermore, the stabilization diagram in the un-truncated order 300 is shown in Figure 2f. It can be seen that the spurious poles increase significantly, when the model order is higher than 150, which partially evidences the noise effect in the Toeplitz matrix.

### 2.3. Cleaning of the Stabilization Diagram Using the DBSCAN Algorithm

**Figure 3:**
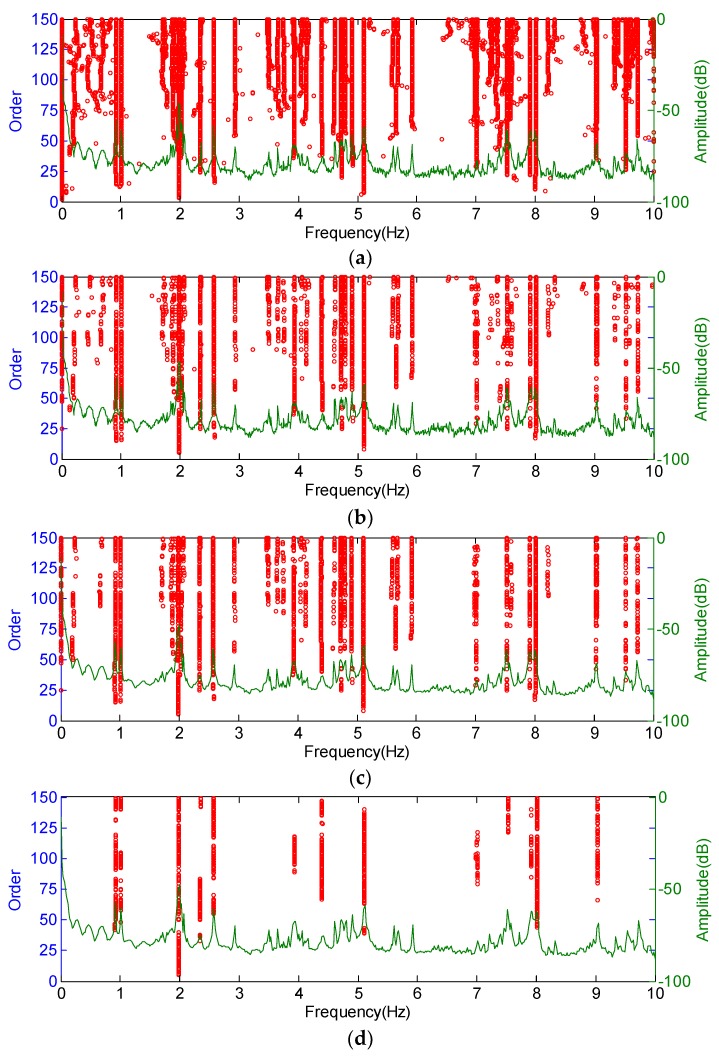
Original stabilization diagram, cleaned stabilization diagram, and stabilization diagram, after the elimination of noise poles by DBSCAN: (**a**) original stabilization diagram; (**b**) cleaned stabilization diagram; (**c**) stabilization diagram, after the removal of noise poles by applying the DBSCAN procedure only to frequency; and (**d**) stabilization diagram, after removing noise poles by applying the DBSCAN procedure to modal frequency, modal damping, and modal shape.

As shown in Figure 3a, the stabilization diagram contains two parts: the power spectral density (PSD) and the identified poles in a different order. The poles generally form a stable axis falling close to the peak of the PSD. The scattered poles are more likely to be mathematical ones, as the physical modes stay within the consistent modal parameters in the consecutive model orders.

Therefore, the poles are labeled as stable if the relative differences in the modal frequency ($\delta_{f}$), modal damping ratio ($\delta_{\varepsilon }$), and *MAC* value between the poles of models with consecutive orders are below the following threshold values:

(11) $$\delta_{f}=1-\frac{f_{i}}{f_{i-1}}<2\%$$

(12) $$\delta_{\varepsilon }=1-\frac{\varepsilon_{i}}{\varepsilon_{i-1}}<5\%$$

(13) $$\delta_{M}=1-MAC(i,i-1)=1-\frac{{\left|\phi_{i}{}^{T}\phi_{i-1}\right|}^{2}}{\phi_{i}{}^{T}\phi_{i}\phi_{i-1}{}^{T}\phi_{i-1}}<5\%$$

where *i* denotes to the current model order, and *i*−1 is the former model order. $f_{i},\;\varepsilon_{i},\;\phi_{i}$ are the *i*th modal frequency, modal damping ratio, and model shape, respectively.

The MPC is an index that is used to describe the modal phase collinearity of the poles [17]. If the structure is proportionally damped, the mode shape vector should lie on a straight line in the complex plane, and the identified mathematical poles would have a low MPC value:

(14) $$MPC(\phi_{i})=\frac{Re{\left(\phi_{i}\right)}^{T}Re(\phi_{i})+\frac{1}{\varsigma }Re{\left(\phi_{i}\right)}^{T}Im{\left(\phi_{i}\right)}^{T}((2\varsigma^{2}+2){sin}^{2}(\theta )-1)}{Re{\left(\phi_{i}\right)}^{T}Re(\phi_{i})+Im{\left(\phi_{i}\right)}^{T}Im(\phi_{i})}$$

(15) $$\varsigma =\frac{Im{\left(\phi_{i}\right)}^{T}Im(\phi_{i})-Re{\left(\phi_{i}\right)}^{T}Re(\phi_{i})}{2Re{\left(\phi_{i}\right)}^{T}Im(\phi_{i})}$$

(16) $$\theta =arctan(\left|\varsigma \right|+sign(\varsigma )\sqrt{1+\varsigma^{2}})$$

where $\;\phi_{i}$ is the *i*th model shape, and *Re*() and *Im*() represent the real and imaginary parts, respectively.

First, the poles with identified modal parameters that do not satisfy Equations (11)–(13) are labeled as spurious ones and are cleaned. Subsequently, the poles with unrealistic damping ratios (ε>10%) and mode shape (MPC<97%) are deleted. Compared with Figure 3a, some local abnormal poles are partially cleaned in Figure 3b. However, unstable poles are still noted, fox example, the poles whose frequencies are below 0.90 Hz in the range from 2.6 Hz to 3.8 Hz and in the scope from 5.3 Hz to 6.3 Hz, etc. Therefore, the designed DBSCAN algorithm is introduced to solve this issue.

Compared with *k*-means clustering and hierarchical clustering methods, the DBSCAN algorithm has a better performance in removing the interference of noise and clustering, without setting the initial value [18]. In this paper, DBSCAN is introduced to cluster the poles, based on modal frequency, damping ratio, and mode shape. In particular, an efficient parameter optimization method, based on the Euclidean distance, is developed to determine a radius *r* and a threshold value minpts to clean the spurious poles.

In a stabilization diagram, regarding a given pole *p* with a specified radius *r*, if the number of neighboring poles within the radius *r* is less than the threshold value minpts, such a pole will be defined as a spurious one. Otherwise, it would be labeled as a stable pole:

(17) $$N_{\varepsilon }(P)=\{q|q\in P,E_{p,q}\leq r\}\geq minpts$$

where *P* represents the set of poles around the given pole *p* within the range of the specified radius *r*, and *q* is one of them. $E_{p,q}$ is the Euclidean distance of the modal parameters between pole *p* and the neighboring poles *q* within the scope of *P*, and $N_{\varepsilon }(P)$ denotes the number of poles satisfying this equation.

In order to optimize the threshold value minpts and the radius *r*, the matrix of the Euclidean distance $E_{d}$ is calculated. It is formed by the Euclidean distance between a certain pole *p* and all other poles in the stabilization diagram (Equation (18)), which reflects the global distances between different poles. Secondly, the column vector of the matrix $E_{d}$ is sorted in ascending order to obtain the matrix $S_{d}$ (Equation (19)). Each column vector of matrix $S_{d}$ characterizes the distribution of the distance between a given pole and the other poles in the stabilization diagram. Finally, the ${minpts}^{\mathrm{th}}$ row of matrix $S_{d}$ is sorted in increasing order, and the vector $SS_{minpts}$ is defined as Equation (20).

(18) $$E_{d}=\left[\begin{array}{l} 0\;e_{1,2}\;\ldots \;e_{1,n}\\ e_{2,1}\;0\;\ldots \;e_{2,n}\\ \ldots \\ e_{n,1}\;e_{n,2}\;\ldots \;0 \end{array}\right]$$

(19) $$S_{d}=\left[\begin{array}{c} 0\;0\;\ldots 0\\ s_{2,1}\;s_{2,2}\;\ldots \;s_{2,n}\\ \ldots \\ s_{n,1}\;s_{n,2}\;\ldots \;s_{n,n} \end{array}\right]$$

(20) $$SS_{minpts}=Sort[S_{minpts,1},S_{minpts,2},\ldots ,S_{minpts,n}]$$

where *n* is the number of all poles in the stabilization diagram, the element $e_{i,j}$ in the *i*th row and the *j*th column of the matrix $E_{d}$ represent the Euclidean distance between the *i*th pole and the *j*th pole, and the element $s_{i,j}$ in the *i*th row and *j*th column of the matrix $S_{d}$ is the Euclidean distance between the *i*th pole and its *j*th nearest pole.

The elements of vector $SS_{minpts}$ are sorted in increasing order, and each element represents the Euclidean distance between every pole and its ${minpts}^{\mathrm{th}}$ nearest pole. Compared with the stable poles, the distribution of spurious poles is more isolated. This means that the distance between the spurious pole and the other pole is relatively larger than that with the stable pole. Therefore, when the value of the element in the vector $SS_{minpts}$ increases suddenly, the corresponding distance should be defined as the optimal radius *r*.

It should be emphasized that every pole in the stabilization diagram consists of three types of modal information: modal frequency, modal damping ratio, and normalized mode shape. Therefore, the procedures of Equations (17)–(20) will be used to clean the spurious poles based on different threshold values of minpts and radius *r* for modal frequency, modal damping ratio, and normalized mode shape. First, poles with abnormal frequencies are defined as noise, and the stable poles are clustered into different groups. Subsequently, DBSCAN with automated parameter optimization is again applied to the poles on the basis of the damping ratio, and the mode shape evaluated by the quadratic sum of the normalized mode shape (*QSNMP*):

(21) $$QSNMP=\phi^{T}\phi$$

Finally, a cleaned stabilization diagram will be obtained, after discarding those spurious poles that do not comply with Equation (17).

Generally, it is assumed that the number of stable poles in a modal order should be greater than 10% of the selected modal order. Thus, the *minpts* for the modal frequency in the DBSCAN procedure is defined as 10% of the selected modal order, as defined in Equation (22):

(22) minptsf=10%×e

where *minptsf* is the defined *minpts* value for the DBSCAN algorithm, based on the modal frequency, and *e* is the selected model order, according to Equations (8)–(10).

**Figure 4:**
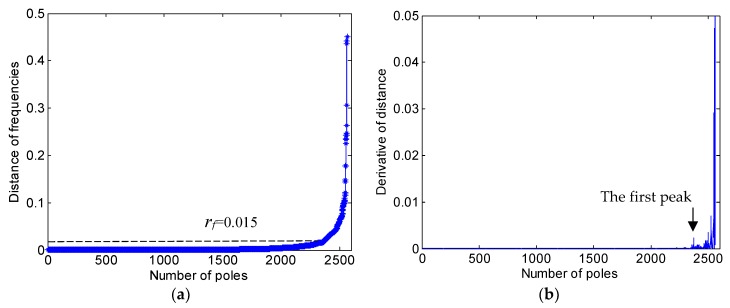
Determination of the $r_{f}$: (**a**) the vector $SS_{15}$; and (**b**) the first peak of the derivative of distance.

For example, with regard to the stabilization diagram in Figure 3b, the selected system model order *e* = 150 and the *minptsf* = 15. The matrix $SS_{15}$, according to Equation (20), and also the 15th row of the matrix $S_{d}$ is plotted in Figure 4a. When the element value of $SS_{15}$ increases suddenly, spurious poles are more likely to appear. The increasing speed of the element value of $SS_{15}$ can be evaluated by the derivative of the sorted elements, as shown in Figure 4b. The first clear peak indicates the appearance of an isolated spurious pole. Thus, the radius of frequency clustering $r_{f}$ should be taken as the corresponding frequency of the Euclidean distance, corresponding to the pole value, which is determined by the first peak in the derivative of the distance. As shown in Figure 4b, the first peak appears at around 2300. The corresponding radius of the DBSCAN algorithm in frequency $r_{f}$ was 0.015 Hz, corresponding to pole 2300 in Figure 4a.

**Figure 5:**
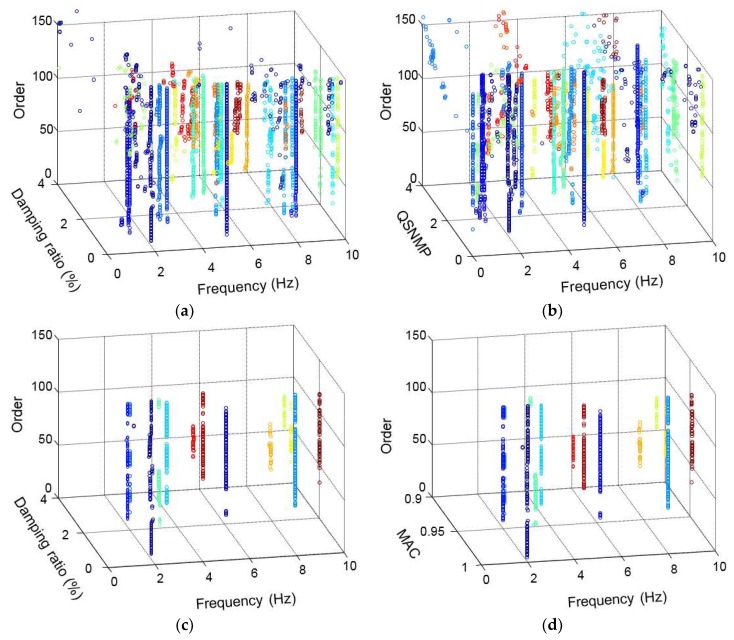
3D stabilization diagram, created by applying the DBSCAN algorithm: (**a**) poles with different damping ratios, after applying the DBSCAN only based on frequency; (**b**) poles with different QSNMPs, after applying the DBSCAN only based on frequency; (**c**) poles after applying the DBSCAN, based on both the modal frequency and damping ratio; (**d**) poles after applying the DBSCAN, based on the modal frequency, damping, and mode shape.

After applying the DBSCAN algorithm based on the modal frequency, the stabilization diagram is further cleaned, as shown in Figure 3c. Compared with Figure 3b, more poles with abnormal frequencies are eliminated, and the remaining poles are clustered in different groups (in the current case, the poles are separated into 36 groups). To exhibit the complete modal information of each pole, a 3D stabilization diagram is constructed, as shown in Figure 5. Figure 5a,b correspond to the 2D stabilization diagram shown in Figure 3c. It is worth noting, from Figure 5a,b, that noise poles still exist with discrete damping ratios and *QSNMP*, even after applying the DBSCAN procedure, based on the modal frequency. In particular, the poles, whose frequencies are below 0.90 Hz in the range from 2.6 Hz to 3.8 Hz and in the scope from 5.3 Hz to 6.3 Hz, are unstable in terms of modal damping or mode shape. Thus, the DBSCAN procedure should be further implemented for the 36 groups of poles based on the modal damping and mode shape evaluated by *QSNMP*, in order to obtain stable poles for all the modal information.

Since the number of poles $g_{i}$ clustered by the frequency-based DBSCAN algorithm varies in different groups, corresponding to different modal orders, the *minpts* and the radius *r* for the DBSCAN procedure, based on the damping ratio and *QSNMP*, should be determined according to the number of poles in each group:

(23) $${minptsd}_{i}=10\%\times g_{i},{minptsq}_{i}=10\%\times g_{i}$$

where $minptsd_{i}$ and $minptsq_{i}$ are the threshold values of the DBSCAN procedure based on the damping ratio and *QSNMP*, respectively. The radius $r_{d_{i}}$ and $r_{q_{i}}$ can be specified by Equations (18)–(20) based on modal damping and *QSNMP*.

After applying both modal damping and *QSNMP*, based on the DBSCAN algorithm, the poles with unstable damping ratios and mode shapes are further excluded. The final 2D and 3D stabilization diagrams are shown in Figure 3d and Figure 5d, respectively. It can be observed, from Figure 5d, that each mode in the different orders has a stable damping ratio and an excellent MAC value, exceeding 95%. The unstable poles, whose frequencies are below 0.90 Hz in the range from 2.6 Hz to 3.8 Hz and in the scope from 5.3 Hz to 6.3 Hz, are further discarded. Finally, the modal parameters in different model orders are identified automatically by clustering the poles in each group.

## 3. Validation of the Automated Modal Analysis Algorithm with an Arch Bridge

In this section, a numerical model and modal experiment on the Rainbow Bridge are used to validate the AMA algorithm proposed in Section 2.

### 3.1. Introduction of the Rainbow Bridge

**Figure 6:**
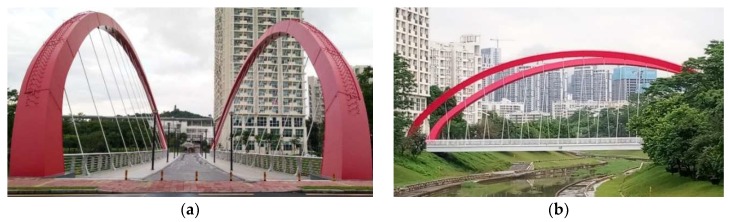
Front view and lateral view of the Rainbow Bridge: (**a**) front view; (**b**) lateral view.

The Rainbow Bridge (Figure 6) over the Dasha River in Shenzhen is located between the Peking University Shenzhen Graduate School and the gymnasium of University Town. The arch bridge is composed of three main parts: two arches, with an inclination angle of 14.4°, 34 pre-stressed cables, and a deck supported by two box girders.

The numerical model of the Rainbow Bridge is established by multiple element types, based on the ANSYS environment. The beam element with shear deformation is used in the simulated arch, while the tension-only link element is applied for the cables. In order to obtain more accurate solutions, the deck is considered as an assembly of shell elements. According to the equivalent stiffness of the pot rubber bearing, the spring elements, with a constant of 1.1E9 N/m, are implemented at the abutment.

The ambient vibration modal experiment is carried out on the bridge deck. As it is difficult to install massive accelerometers on an inclined arch, the physical mode of the arch is verified by three fixed lateral accelerometers for long-term monitoring (Section 4) and numerical simulation.

**Figure 7:**
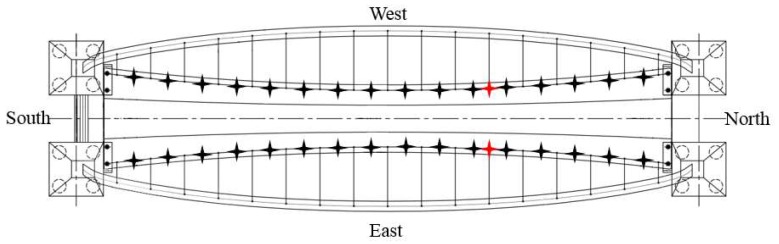
Measured points in the modal test.

To obtain both the bending and torsional modes with a higher spatial resolution, an ambient vibration experiment was performed on both sides of the deck by a multi-reference point method. As shown in Figure 7, the 32 black stars are the test points, while the two red stars are the reference points. A total of 32 test points is divided into 16 setups. In each setup, the bridge responses are measured, with an initial sampling frequency of 2048 Hz lasting 15 min, and these are processed with a low-pass anti-aliasing filter of 10 Hz and are further down-sampled to 20 Hz by cubic spline interpolation.

### 3.2. Validation of the Automated Modal AnalysisAlgorithm

**Figure 8:**
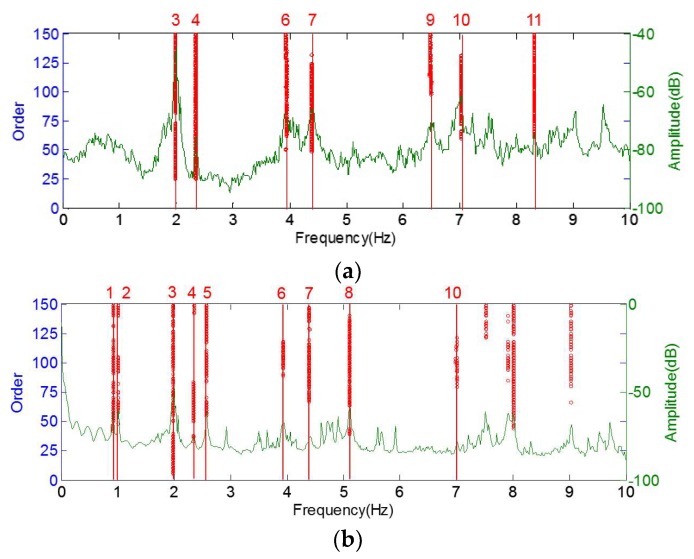
Cleaned stabilization diagram of the deck and the arch using the DBSCAN algorithm: (**a**) stabilization diagram obtained from the acceleration signals in the deck; (**b**) stabilization diagram obtained from the acceleration signals on the arch.

**Figure 9:**
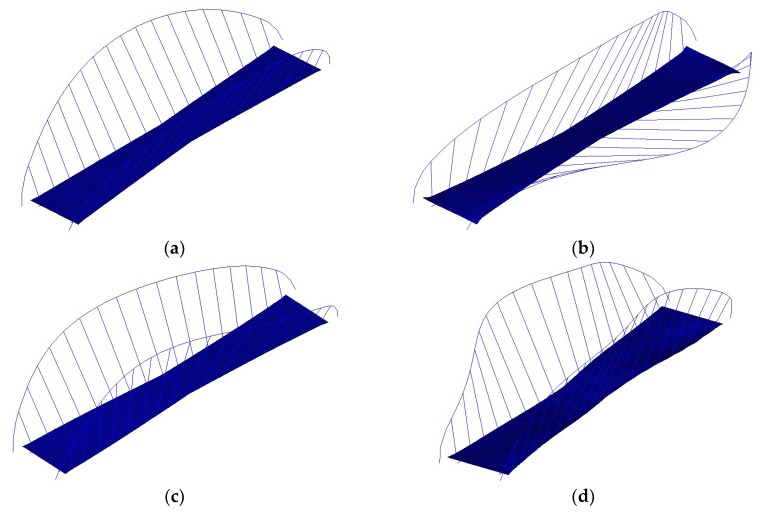
Mode dominated by the arch: (**a**) the 1st mode (0.93 Hz); (**b**) the 2nd mode (0.99 Hz); (**c**) the 5th mode (2.50 Hz); (**d**) the 8th mode (5.22 Hz).

**Figure 10:**
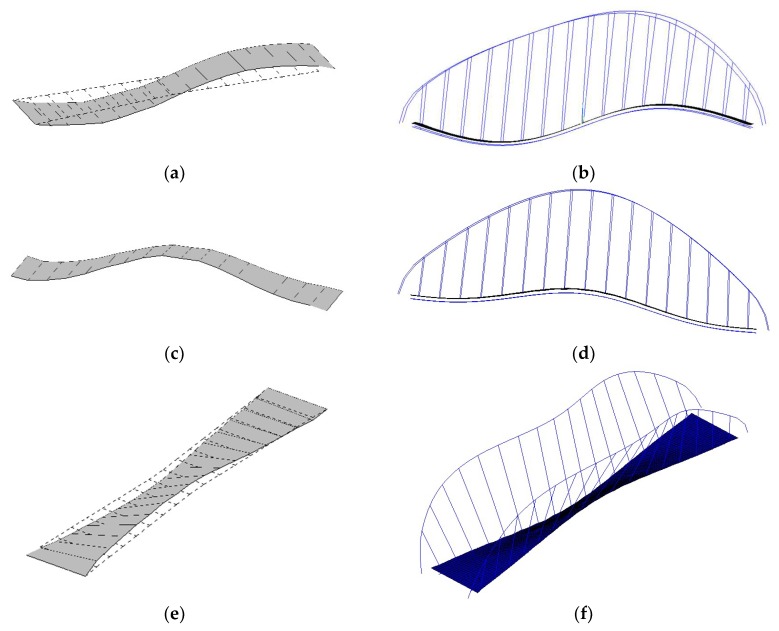

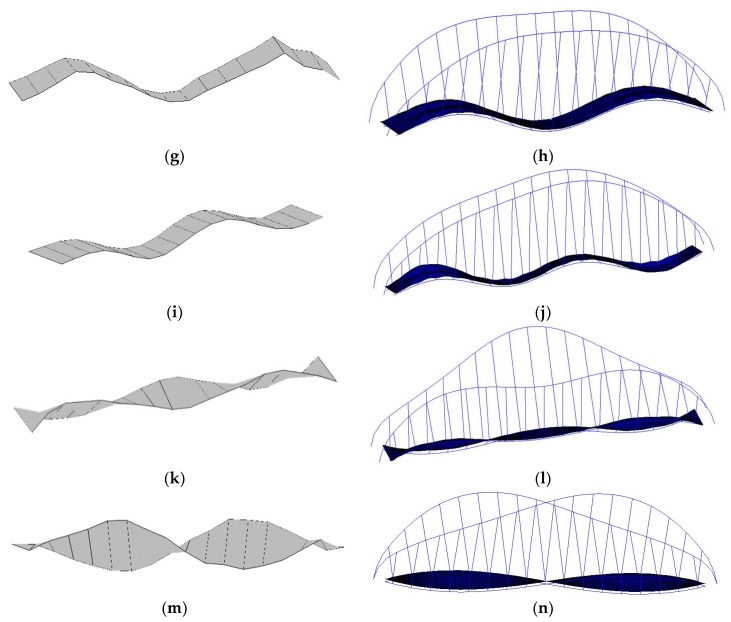
Mode coupled by the deck and arch: (**a**) the 3rd mode identified by SSI-COV (1.99 Hz); (**b**) the 3rd mode calculated by ANSYS (2.00 Hz); (**c**) the 4th mode identified by SSI-COV (2.34 Hz); (**d**) the 4th mode calculated by ANSYS (2.56 Hz); (**e**) the 6th mode identified by SSI-COV (3.94 Hz); (**f**) the 6th mode calculated by ANSYS (3.81 Hz); (**g**) the 7th mode identified by SSI-COV (4.39 Hz); (**h**) the 7th mode calculated by ANSYS (4.34 Hz);(**i**) the 9th mode identified by SSI-COV (6.48 Hz); (**j**) the 9th mode calculated by ANSYS (6.54 Hz); (**k**) the 10th mode identified by SSI-COV (7.03 Hz)); (**l**) the 10th mode calculated by ANSYS (7.32 Hz); (**m**) the 11th mode identified by SSI-COV (8.32 Hz); (**n**) the 11th mode calculated by ANSYS (8.64 Hz).

The bridge responses are further analyzed by the AMA algorithm, as stated in Section 2. The modal parameters of the physical mode, identified by the ambient vibration experiment and calculated by the numerical simulation, are listed in Table 1. Furthermore, the visualized mode shapes identified by the LabVIEW toolkit [26], are shown in Figure 8, Figure 9 and Figure 10.

It can be seen from Table 1 that the frequency differences between the identified and calculated results are less than 5%, except for the 4th mode, and the MAC values between them exceed 97%. This means that the numerical simulation and field modal experiment are well matched.

It is interesting to note, from both the numerical simulation and ambient vibration experiment, that some modes are dominated by the arch, while some modes are coupled by both the arch and the deck. As shown in Figure 8 and Table 1, the 1st, 2nd, 5th and 8th modes can only be recognized in the stabilization diagram of the arch. The corresponding mode shapes, calculated by ANSYS, are presented in Figure 9. They are also the 1st (both symmetry and anti-symmetry) to the 3rd transversal modes of the arch, respectively.

Generally, the vibration of the arch and deck are coupled. As shown in Figure 10, the 3rd, 4th, 6th, 7th and 10th modes are coupled by the vertical mode of the deck and transversal mode of the arch, which could be identified in the stabilization diagram of the deck and the arch, while the 9th and 11th modes are only observed in the stabilization diagram of the deck. A possible reason for such a phenomenon may be that the arch vibration in Figure 10j is along the longitudinal direction, which cannot be captured by lateral accelerometers. In summary, all the spurious modes are eliminated, and the identified modal parameters are reliable when using the AMA algorithm. The tracking of the modal frequencies and their application to structural change detection are exhibited in Section 4.

## 4. The Continuous Dynamic Monitoring System and Tracking of Long-Term Modal Frequency

In this section, the continuous dynamic monitoring system is introduced, and the frequencies of different bridge modes are tracked by an AMA, implemented with the DBSCAN algorithm to track the structural change during the construction phase. Subsequently, the temperature effect on frequency under the operation phase is eliminated by ANN, and the abnormal detector (*AD*) is established by the Euclidean distance of the error matrix.

### 4.1. The Continuous Dynamic Monitoring System

**Figure 11:**
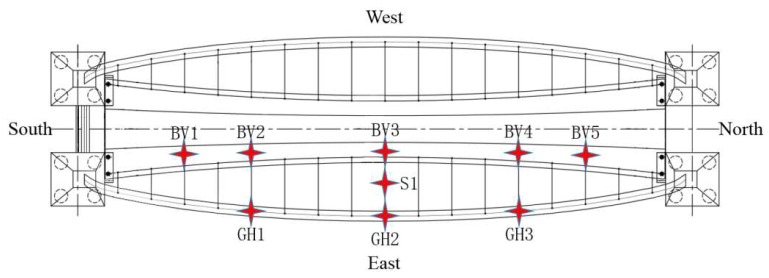
Distribution of the accelerometers.

The Rainbow Bridge began construction in November 2016 and was finished in June 2017. In order to monitor structural long-term behavior, a continuous dynamic monitoring system was implemented on this bridge, and it began to work on 1 March 2017. The vertical vibration of the deck and the transversal vibration of the arch were designed to be the main monitoring objects, according to the modal analysis of the FE model. In order to ensure that each physical mode was identified, accelerometers are selected and installed at the corresponding position of the red stars in Figure 11. BV1–BV5 represents the five vertical magneto-electric accelerometers on the deck, while GH1–GH3 show the position of the three horizontal magneto-electric accelerometers on the arch. Meanwhile, a piezoelectric accelerator is applied to monitor the vibration of the longest cable (S1).

**Figure 12:**
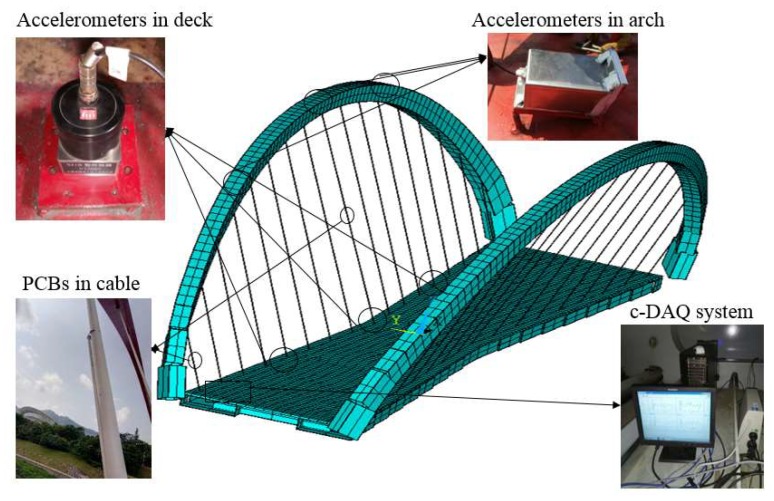
The continuous dynamic system.

Figure 12 shows the continuous dynamic system, providing high-quality data. Acceleration signals in both the deck and arch are continuously acquired, with a sampling ratio of 2000 Hz, and they are connected to a low-pass anti-aliasing filter of 10 Hz, as well as being down-sampled to 20 Hz by the spline interpolation. The cable acceleration signal is applied with a low-pass filter of 25 Hz, and then down-sampled to 50 Hz. All acceleration signals acquired within 10 min are stored in a local computer in a nonstop manner. Furthermore, a continuous transmission toolkit is developed to automatically deliver the acceleration signals from the bridge to the laboratory.

### 4.2. Tracking of the Long-Term Modal Frequency

The continuous monitoring system started to operate on 1 March 2017. Until 31 July 2018, signals taken during a time-span of 320 days are available, although some days are missing due to the instability of the power supply in the construction phase. Fortunately, several interesting events were recorded by the continuous monitoring system.

As described in Section 3, some modes are dominated by the transversal vibration of the arch, while others recoupled with both the transversal arch and vertical deck vibrations. Thus, the physical modes are divided into two categories to track the long-term modal frequencies.

**Figure 13:**
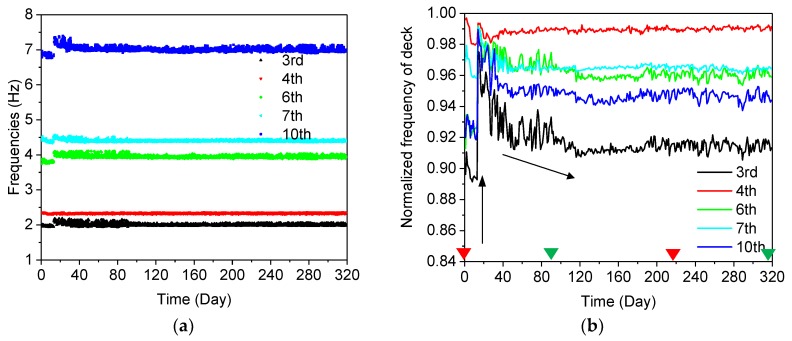
Modal frequencies of the arch lateral and deck vertical vibrations from 1 March 2017 to 31 July 2018: (**a**) the long-term frequencies of the coupled modes; (**b**) the average normalized frequencies of the coupled modes. (The two triangle marks in green: the 90th day (15 August 2017) and the 320th day (31 July 2018); The two triangle marks in red: the 1st day (1 March 2017) and the 220th day (1 March 2018)).

As shown in Figure 13a, on the 17th day (5 April 2017), an obvious jump appeared in the 3rd, 4th, 6th, 7th and 10th modes, which is due to the modest coupling of both the arch and deck vibrations. Afterwards, the frequencies decreased gradually until the 90th day (15 August 2017). In order to illustrate the variation tendency in a better way, the average normalized frequencies were introduced:

(24) $$f_{j}=\frac{\sum_{i=24\times j-23}^{24\times j}f_{i}}{24\times {\left(f_{i}\right)}_{max}},(j=1,2,\ldots ,320)$$

where $f_{i}$ is the number of identified frequencies, and *i* represents the total number of frequencies, which was 24 × 320 = 7680 in this case. $f_{j}$ represents the average normalized frequencies in each day.

**Figure 14:**
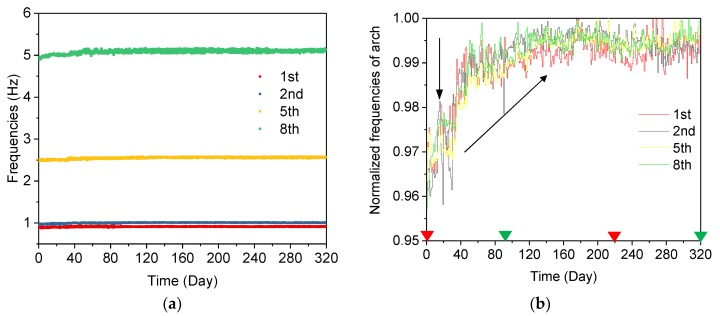
Frequencies of the lateral arch and vertical deck from 1 March 2017 to 31 July 2018: (**a**) the long-term frequencies of the modes dominated by the arch; (**b**) the average normalized frequencies of the modes dominated by the arch. (The two triangle marks in green: the 90th day (15 August 2017) and the 320th day (31 July 2018); The two triangle marks in red: the 1st day (1 March 2017) and the 220th day (1 March 2018)).

**Figure 15:**
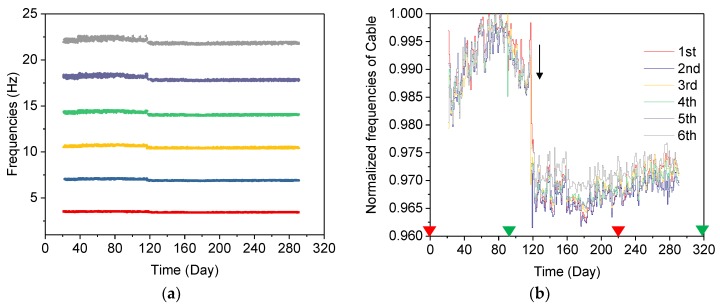
Frequencies of the longest cable (cable 9) from 1 March 2017 to 31 July 2018: (**a**) the long-term frequencies of the cable; (**b**) the average normalized frequencies of the cable.

The calculated normalized frequencies are shown in Figure 13b. In order to illustrate the annual variation tendency of the frequency clearly, four time data notes (four triangles in both red and green) are labelled in Figure 13b, Figure 14b and Figure 15b for the arch-deck coupled mode, deck mode and cable mode. The two green triangle marks are the 90th day (15 August 2017) and the 320th day (31 July 2018), respectively. The two red triangle marks are the 1st day (1 March 2017) and the 220th day (1 March 2018).

For all identified coupled modes, it can be observed that the frequencies jump on the 17th day and then decrease until the 90th. In the period from the 90th day (15 August 2017) to the 320th day (31 July 2018), during a time-span of one year, the frequencies in different modes fluctuate regularly due to temperature. 

The identified frequencies of the 1st, 2nd, 5th, and 8th modes are tracked in Figure 14a, and the corresponding average normalized frequencies are plotted in Figure 14b. It is interesting to note that the modal frequencies dominated by the arch show the opposite behavior to those with the coupled modes. Comparing Figure 13b with Figure 14b, it can be observed that the average normalized frequencies in Figure 13b keep rising until the 17th day, while the coupled modes decrease in this period, as shown in Figure 14b. Then, a sudden change, but with a different tendency, is observed in both Figure 13b and Figure 14b, and subsequently, the frequencies of the arch-dominated mode keep rising until the 90th day. In the period from the 90th day to the 320th day, an opposite fluctuation trend is observed in the arch-dominated mode, compared with the tendency in the arch-deck coupled mode.

Cable force can be evaluated continuously by tracking the frequency variation [27,28,29]. The cable frequencies of each order are integer multiples of the fundamental frequencies, as shown in Figure 15a. The average normalized frequencies of the monitored cable are displayed in Figure 15b. It can be seen that the normalized frequencies rise during the period from the 20th day to the 80th day and decrease slowly from the 80th to the 120th day, while a sudden drop occurs on the 120th day (9 November 2017). Afterwards, the normalized frequencies oscillate with temperature.

**Figure 16:**
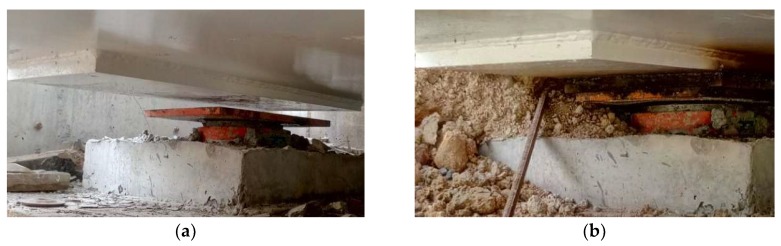
The change of the boundary condition: (**a**) before the 17th day (5 April 2017); (**b**) after the 17th day (5 April 2017).

**Figure 17:**
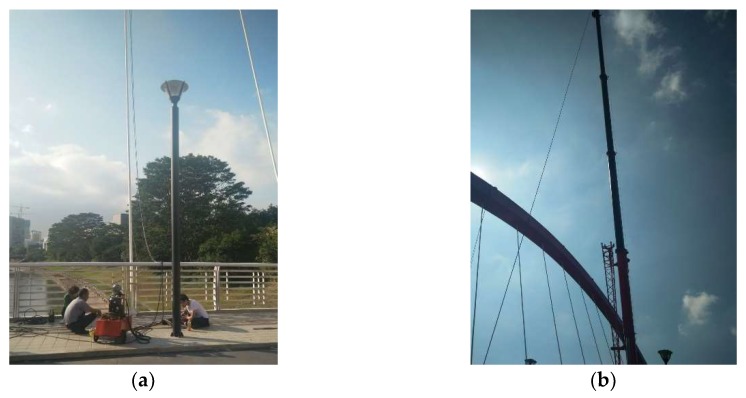
The adjustment of cable forces (cable 8 and 10): (**a**) before the 120th day (9 November 2017); (**b**) after the 120th day (9 November 2017).

The important events in the bridge construction phase may partially explain the variation of frequencies of different structural modes shown in Figure 13b, Figure 14b and Figure 15b. From the 1st day to the 17th day, the concrete deck was cast on the bridge, leading to decreasing frequencies in the coupled mode and increasing frequencies in the arch-dominated mode. Meanwhile, one of eight bearings was in a state of suspension, without supporting the bridge box girder (Figure 16a), due to an unexpected construction error. On the 17th day, the bearing was repaired and the boundary condition changes significantly, resulting in sudden changes of the tracked frequencies of both the deck and arch, as shown in Figure 13b and Figure 14b. On the 120th day (9 November 2017), the cable force of the 2nd, 8th and 10th cables reduced by 20, 10 and 20 tons, respectively, and the 13th cable force increased by 20 tons, ensuring that all cable forces reached a designated value (Figure 17). The adjustment in the cable force resulted in the sudden drop in the frequencies of the 9th cable (the longest cable) on the 120th day (9 November 2017). It can be concluded that variations of the modal frequency, tracked by the AMA based on DBSCAN algorithm can reveal structural changes in the construction phase.

### 4.3. Removal of the Temperature Effect on Modal Frequency

**Figure 18:**
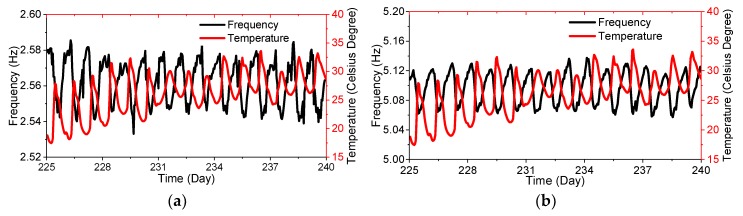
The relationship between the temperature and frequencies: (**a**) the 5th mode; (**b**) the 8th mode.

The temperature is reported to have a significant influence on the frequencies. After the 120th day, the bridge is open for operation. The correlation between temperature and tracked frequencies of the arch are shown in Figure 18. Under operation conditions, daily temperature changes resulted in about 2% frequency fluctuations in one day, while the maximum frequency fluctuation, caused by the annual temperature differences, reaches about 4.5% (Figure 14). Therefore, the feature that is only sensitive to structural change should be extracted by removing the non-linear temperature effects on frequencies.

The polynomial regression model (PR) [30] and artificial neural networks (ANN) [31] are the main mathematic models fitting non-linear relationships. An optimal PR model can be obtained by the generalized least square method. Theoretically, the rising model order of PR can decrease the fitting errors, but it also leads to an over-training problem. Thus, the Akaike information criterion (AIC) is introduced to determine the optimal model order of the PR method. The detailed algorithm in [10] can be consulted. In this paper, a four-order PR model is used to fit the nonlinear relationship between the temperature and arch frequency.

The ANN algorithm is designed to simulate the mechanism of neural networks in the human brain. The independent neurons, representing mathematical functions, are interconnected in networks to forecast outputs. The networks contain three parts: one input layer, hidden layers (there can be more than one), and one output layer. Each layer node has the former layer node values multiplied by the associated weights, and processes them with a certain function:

(25) $$S_{x,i}=f(\sum_{i}S_{x-1,i}\cdot W_{x,i})$$

where f() represents the processing function (normal sigmoid function: $y=\frac{e^{x}-e^{-x}}{e^{x}+e^{-x}}$), $S_{x,i}$ and $W_{x,i}$ are the value and weights of the $i^{th}$ neuron in the $x^{th}$ layer, respectively.

A back-propagation algorithm is performed to train the neural networks. Along with the training procedure, the weights of each node in each layer will approximate accurate values. The initial values of weight *W* are set to random numbers (normally, −0.1 to 0.1). After multiple iterations, the errors *E* between the output values *Y* and target value *T* are calculated. The errors *E* will propagate backward by changing the weight (*dW*):

(26) $${dW}_{x,i}=LR\times E_{x,i}\times f^{\prime }(S_{x,i})\times Y_{x,i}\;(\mathrm{for}\;\mathrm{the}\;\mathrm{weight}\;\mathrm{connected}\;\mathrm{to}\;\mathrm{output}\;\mathrm{neurons})$$

(27) $${dW}_{x-1,i}=LR\times E_{x-1,i}\times f^{\prime }(S_{x,i})\times Y_{x,i}\;(\mathrm{for}\;\mathrm{the}\;\mathrm{weight}\;\mathrm{connected}\;\mathrm{to}\;\mathrm{output}\;\mathrm{neurons})$$

where *LR* is the learning rate, *S_x_*_,_*_i_* is the value calculated by Equation (26), *Y_x_*_,*i*_ is the output value of neurons, *x* represents the layer number, *I* represents the neuron number, and *j* represents the weight number.

The weights are optimized for the next iteration by:

(28) $$W_{n+1}=W_{n+1}+{dW}_{n}$$

In the current research, the temperature is taken as the input variable, while the arch frequency in different orders is set as the output variable. One hidden layer with two neurons is set for the curve fitting. In order to prevent over-training of the network, data in both the input and output layers are split into two groups. The first group consists of odd data points (${dp}_{n}(n=1,3,5,\ldots )$) and is used for training the model, while the other group includes even data points (${dp}_{n}(n=2,4,6,\ldots )$) and is applied to test the trained model.

**Figure 19:**
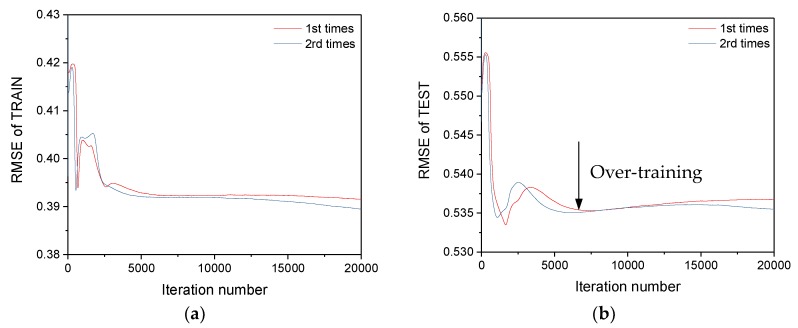
The RMSE of the training and testing groups: (**a**) the RMSE of training; (**b**) the RMSE of testing.

The iteration number of the ANN model is first determined to avoid the overtraining problem. The root mean square of error (RMSE) between the predicted frequency and the tracked frequency is used to evaluate the performance of the ANN model. Over-training occurs when the RMSE of training decreases and the RMSE of testing increases. The training procedure is executed with two different initial values of weight *W*, ranging from –0.1 to 0.1. As shown in Figure 19a, the RMSE for both training models, with different initial values, decreases with increasing iteration numbers. When the iteration number approximates 7000, the RMSE of both testing groups starts to rise, which suggests over-training of the models. Thus, the iteration number of the proposed ANN model is set as 7000.

**Figure 20:**
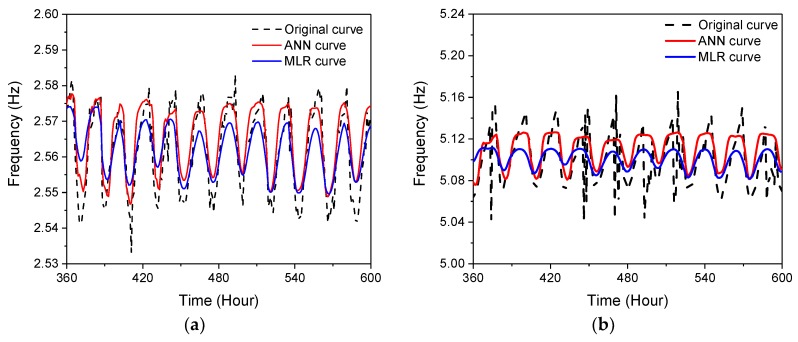
Comparison of ANN and PR in the fitting of identified frequencies: (**a**) the 5th mode; (**b**) the 8th mode.

The performance of the removal of the temperature effect based on the ANN and PR models is compared in Figure 20. It is evidenced that the predicted frequency based on the ANN model is closer to the tracked frequency, indicating that the ANN model has a better fitting capacity than the PR model.

**Figure 21:**
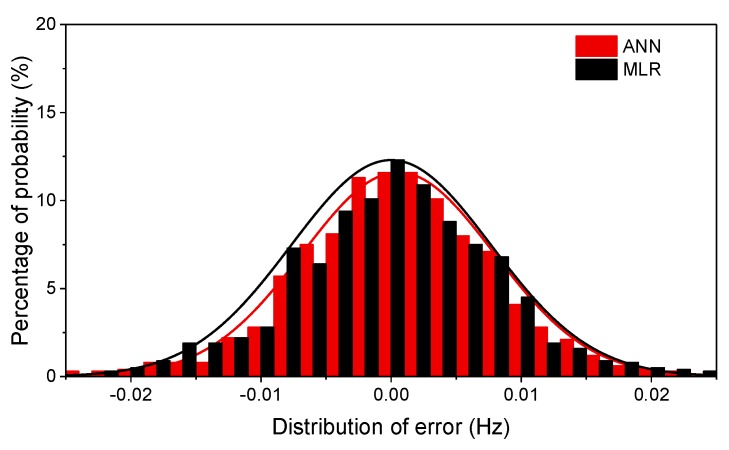
The distribution of error vectors of the ANN and PR models for the 5th model.

Figure 21 shows the distributions of error between the predicted and tracked frequency based on both the ANN and PR models. Table 2 lists the RMSE of both models. The RMSE values produced by the ANN model are smaller than those computed by PR, suggesting a better fitting performance of the ANN model. Therefore, the optimal ANN model is chosen to remove the temperature effect and establish the health index for the arch bridge.

Error vectors between the predicted and tracked frequency are regarded as only sensitive to structural change since the temperature is effectively removed:

(29) $$\varepsilon_{i}=f_{i}-{\tilde{f}}_{i}$$

where $f_{i}$ is the $i^{th}$ order identified frequency while ${\tilde{f}}_{i}$ is the $i^{th}$ order frequency estimated by ANN, and $\varepsilon_{i}$ represents the $i^{th}$ error vector. 

Establishing the error matrix $E=[\varepsilon_{1}\ldots \varepsilon_{i}\ldots \varepsilon_{k}]$ and defining the anomaly detector (***AD***) to display the occurrence of structural change gives [32]:

(30) $$AD=\sqrt{\left\|E\right\|}$$

(31) CL=μ

(32) UCL=μ+ασ

where *CL* is the central limit, while *UCL* and *LCL* represent the upper and lower central limit, respectively. μ and σ are the mean value and standard deviation of ***AD*** in the “health state”. Taking as 3 means a confidence level of 99.7%.

**Figure 22:**
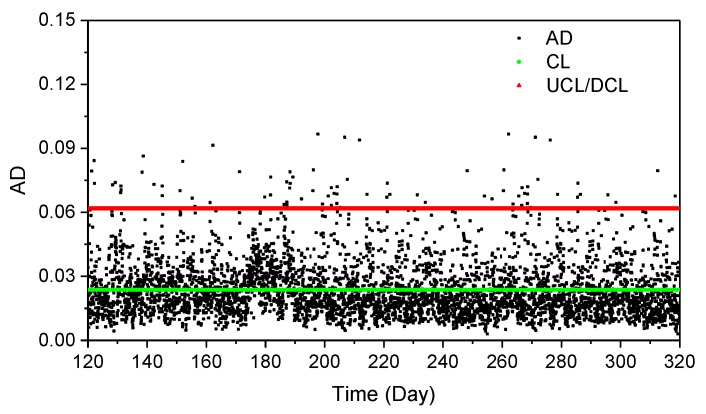
The abnormal detector, *CL*, *UCL* and *DCL* during operational conditions.

Figure 22 shows the ***AD***, calculated by removing the temperature effect on arch frequency, based on the ANN model. It can be observed that the temperature effect is efficiently removed, and the ***AD*** serves as the health index for the arch bridge under operational conditions.

## 5. Conclusions

This paper proposes an automated modal analysis (AMA) algorithm, based on the system model order determination and the DBSCAN method. By applying the algorithm, all the physical modes are identified, and the corresponding modal parameters are extracted. Furthermore, a continuous monitoring system in an arch bridge is implemented to track the long-term frequency, based on the proposed AMA. The main conclusions can be drawn as follows:

(1) The stabilization diagram, identified by the acceleration signals of the arch and deck, shows two different patterns. One was dominated by the arch, and the other was coupled by both the arch and deck. The identified modal parameters of both patterns coincided well with the numerical simulation, which partially validates the correctness of the proposed AMA algorithm.
(2) By applying the AMA algorithm, the long-term modal frequencies of the physical modes from March 2017 to July 2018 were tracked. During the construction phase, several clear fluctuations of the frequencies of the deck, arch and cables are observed. They reflect structural changes, such as modification of boundary conditions and adjustment of cable forces.
(3) Under the operation condition, obvious temperature effects on frequencies are observed. By comparing two nonlinear curve fitting algorithms of both PR and ANN, the latter was proved to be more efficient in eliminating the temperature effect. The *AD* index, extracted from the error matrix of the ANN model, serves as the health index for the bridge under operational conditions.

## Figures and Tables

**Table 1:** Results of the numerical simulation and modal experiment.

Order	FEM (Hz)	SSI (Hz)	Description	Errors	MAC
1	0.93	0.92	1st arch transversal (symmetry)	1.08%	-
2	0.99	1.01	1st arch transversal (anti-symmetry)	1.98%	-
3	2.00	1.99	2nd deck vertical + 2nd arch vertical and transversal (symmetry)	0.50%	0.99
4	2.56	2.34	1st deck vertical + 1st arch vertical and transversal (symmetry)	10.26%	0.99
5	2.50	2.55	2nd arch transversal (anti-symmetry)	1.96%	-
6	3.81	3.94	1st deck torsion + 3rd arch transversal (anti-symmetry)	3.30%	0.98
7	4.34	4.39	3rd deck vertical + 3rd arch transversal (symmetry)	1.10%	0.97
8	5.22	5.11	3rd arch transversal (symmetry)	2.15%	-
9	6.54	6.48	4st deck vertical + arch longitudinal (symmetry)	0.92%	0.98
10	7.32	7.03	3st deck torsion +3rd arch vertical and transversal (symmetry)	4.13%	0.98
11	8.64	8.32	2st deck torsion + arch longitudinal (anti-symmetry)	3.85%	0.99

**Table 2:** The RMSE of errors produced by the ANN and PR models.

	RMSE (Hz)			
	1st Mode	2nd Mode	5th Mode	8th Mode
ANN	0.0056	0.0056	0.0068	0.0075
PR	0.0063	0.0066	0.0078	0.0084
